# Computer-Guided Surgery Can Avoid Prophylactic Endodontic Treatment in Autologous Tooth Transplantation: A 5-Year Follow-Up Case Report

**DOI:** 10.3390/dj12050124

**Published:** 2024-04-25

**Authors:** Luca Boschini, Amerigo Giudice, Michele Melillo, Francesco Bennardo, Francesc Abella Sans, Matteo Arcari, Luigi Tagliatesta

**Affiliations:** 1 Dental Clinic, Ambulatorio Odontoiatrico Boschini, 47922 Rimini, Italy; luca.boschini76@gmail.com; 2 Department of Health Sciences, School of Dentistry, Magna Graecia University of Catanzaro, 88100 Catanzaro, Italy; a.giudice@unicz.it; 3 Department of Clinical and Experimental Medicine, University of Foggia, 71122 Foggia, Italy; drmichelemelillo@gmail.com; 4 Department of Endodontics, Universitat Internacional de Catalunya, Sant Cugat del Vallès, 08195 Barcelona, Spain; franabella@uic.es; 5 Unit of Oral Surgery, Department of Health Sciences, Santi Paolo and Carlo Hospital, University of Milan, 20142 Milan, Italy; matteo.arcari96@gmail.com (M.A.);

**Keywords:** computer-guided surgery, root canal treatment, third molar, tooth autotransplantation

## Abstract

Autotransplantation is a successful technique to replace compromised teeth. This study presents a computer-guided surgical approach for preparing the receiving socket for a mature mandibular third molar donor tooth with a wait-and-see approach instead of prophylactic endodontic treatment. A 42-year-old woman developed root resorption of tooth 3.7. Extraction of 3.7 and autotransplantation of 3.8 was planned, following a 6-week orthodontic phase for periodontal ligament activation and teeth mobilization. Due to the different root morphology between the compromised and donor teeth and the high mandibular bone density, the receiving socket preparation was performed using guided surgery templates. Two surgical splints were designed with a surgical planning software. Tooth 3.7 was extracted, the recipient site was guided-milled, and tooth 3.8 was transplanted into the new socket in approximately one second of extra-alveolar time. The rapidity of the extra-alveolar time facilitated complete healing without resorting to root canal treatment. Five-year radiological control does not show any periapical lesion or root resorption. The surgical procedure for tooth autotransplantation is fundamental: it must be as atraumatic as possible to preserve the periodontal ligament of the tooth and the receiving socket, and the dentist must minimize the extra-alveolar time. Guided surgery is a reliable solution to combine all these aspects.

## 1. Introduction

Autotransplantation is a reliable treatment option for the replacement of irremediably compromised teeth. The improved long-term success rate of this technique is reportedly influenced by several factors, such as the stage of development of the donor tooth and its morphology, the surgical procedure, the extra-alveolar time, the shape and the vascularity of the receiving socket, and the vitality of the periodontal ligament (PDL) cells [1,2]. Autotransplantation reliability has been demonstrated both with mature and immature teeth [3,4]. According to the literature, endodontic treatment of the transplanted tooth with complete root formation is necessary to prevent or halt the development of periodontal or pulp-related diseases. These elements have less potential for pulp healing, and the extra-oral time may be a risk of septic necrosis. In contrast, teeth with incomplete root formation offer the advantage of pulp revascularization and reinnervation, a process closely related to the roots’ developmental stage and to the apical foramen’s dimension [5,6].

Recipient socket preparation is one of the keys to the procedure’s success: it must be as atraumatic as possible to avoid overheating bone during the osteotomy, which may lead to cell death and vascularization reduction, inhibiting the formation of new bone. These considerations are mainly related to bone density, mostly in the mandibular arch. To minimize complications, digital technology has been increasingly used for tooth transplantation to simplify treatment planning and surgery. Using computer-aided rapid prototyping (CARP) models allows the clinician to prepare the recipient site without needing the donor tooth, permitting the maintenance of the PDL cells on its root surface and reducing its extra-alveolar time. Furthermore, surgical planning software enables the design and manufacture of three-dimensional (3D)-printed surgical templates for guided preparation of the receiving socket, like for dental implants [7,8]. Also, a customized osteotome can be made for the preparation of the receiving site to obtain a new socket with the exact shape of the donor tooth; this surgical approach is optimal for the upper arch, where the bone is less mineralized and can be associated with the use of surgical templates [8].

This study aims to report a successful guided surgery in the autotransplantation of a mature lower third molar. This approach resulted in a short extra-alveolar time of the donor tooth, allowing complete healing and avoiding root canal treatment.

## 2. Materials and Methods

This case report has been written according to the Preferred Reporting Items for Case reports in Endodontics (PRICE) 2020 guidelines [9]. The flowchart regarding the PRICE guideline is reported in Table 1. The local ethics committee was consulted and stated that no approval was needed for this case report.

**Figure 1:**
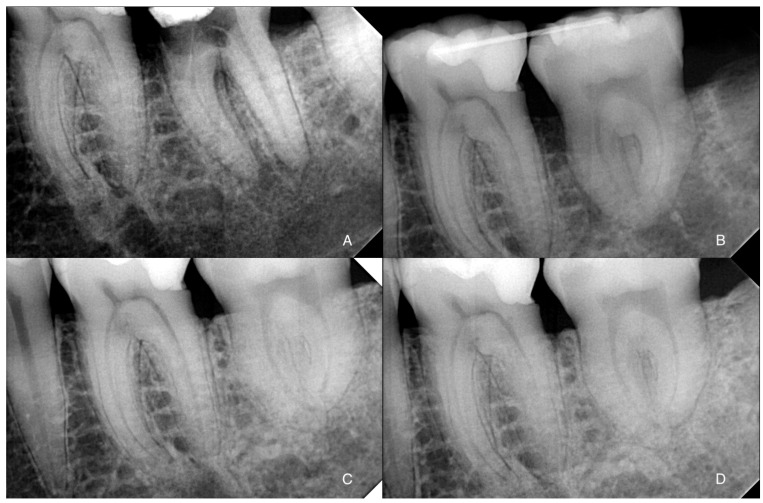
(**A**) X-ray showing the cervical root resorption of tooth 3.7; (**B**) X-ray showing the transplantation of 3.8 in 3.7 position immediately after the surgery; (**C**) X-ray showing the healing at 1-year follow-up; (**D**) X-ray at 3-year follow-up.

A 42-year-old woman presented to our attention with slight pain in tooth 3.7. The clinical examination revealed a mesial cavity developing deeply under the gingiva, compatible with root resorption. This diagnosis was confirmed by X-ray (Figure 1A) and Cone Beam Computerized Tomography (CBCT). The gum was healthy, and thermal vitality tests testified to the vitality of the affected tooth.

Due to the resorption extension and the development of bone tissue inside the cavity up to the pulp chamber, the tooth had to be extracted. Tooth 3.8 was present and in good health, so its autogenous transplant was planned to replace 3.7 as an alternative treatment to implant rehabilitation. The patient approved this solution and signed a specific, informed consent form.

**Figure 2:**
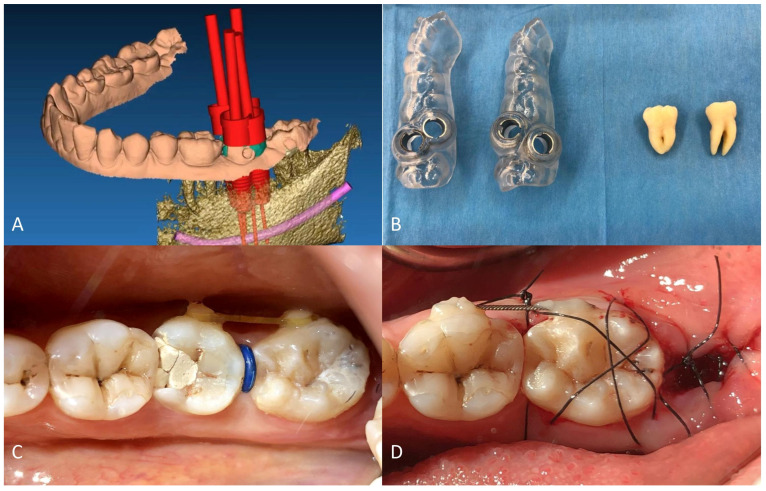
(**A**) Planning of the template simulating the positioning of four dental implants superimposed to the donor tooth virtually moved in the recipient site (in green); (**B**) templates and CARP resin models of both the compromised and donor teeth; (**C**) pre-operative field showing the attachment for the initial orthodontic phase; (**D**) post-operative image with suspended sutures and wire fixing.

Tooth 3.7 had a very different root conformation compared to tooth 3.8. Given the hardness of the mandibular bone, we decided to prepare a surgical guide for the preparation of the recipient site with implant drills. Using a specific software for guided implantology (RealGUIDE™ 5.0, 3DIEMME Srl, Como, Italy), the donor tooth (3.8) was segmented from the CBCT and virtually moved in the alveolar space of tooth 3.7. We designed the surgical guide mimicking the insertion of four implants superimposed on the roots of the donor tooth to prepare the recipient site appropriately (Figure 2A). Through bone-milling with the guides designed by the software, the obtained site would be able to receive the donor tooth. 

Surgical planning resulted in two guides that were 3D-printed after exporting STL files. CARP models of both teeth were printed to evaluate the discrepancy between the milled recipient site and the teeth dimension (Figure 2B).

After this phase, the patient received a 6-week orthodontic pre-treatment to initiate teeth mobilization to simplify the extractions and activate the PDL. Four composite buttons (one buccal and one lingual in each tooth) were applied to the crowns of 3.7 and 3.8. Alternating forces were applied weekly, inserting a chain between the buccal buttons and then on the lingual ones. A separating rubber band was also inserted every week (Figure 2C). 

The patient received instructions for antibiotic therapy (Amoxicillin 1 g tablets, *ii* 1 h before the procedure, and *bds* for one week) and antiseptic rinses (Chlorhexidine 0.1%, *tid* rinses after daily oral hygiene procedures for two weeks, starting two days before surgery).

Immediately before the procedure, the patient rinsed with Chlorhexidine 0.3% mouthwash for one minute, and the lips and perioral tissues were disinfected with a povidone-iodine solution. The anesthetic solution with 4% articaine with epinephrine (1:100,000) was delivered locally through an inferior alveolar nerve block and buccal infiltration. The surgery was performed flapless: tooth 3.7 was extracted through a magneto-dynamic device (Magnetic Mallet, Osseotouch, Varese, Italy). After 3.7 extraction and before 3.8 extraction, the recipient socket was prepared with the surgical guides using a specific guided-surgery implant drill kit (Anyridge, Megagen, Seoul, Republic of Korea). Subsequently, the CARP model of tooth 3.8 was inserted in the recipient socket to check the efficacy of the guided surgical protocol. Tooth 3.8 was extracted using forceps, taking care not to injure the PDL; the forces applied were slow and progressive to tear the collagen ligament fibers gently. The donor tooth was immediately inserted into the recipient site as soon as it was extracted, so the extra-alveolar time was approximately one second. No root surface modifications were performed on the donor tooth before the insertion in the socket. The transplanted tooth was then fixed with suspended sutures and a passive flexible wire bonded with a composite (Figure 1B and Figure 2D). An occlusal adjustment was necessary to reduce the occlusal forces.

## 3. Results

**Figure 3:**
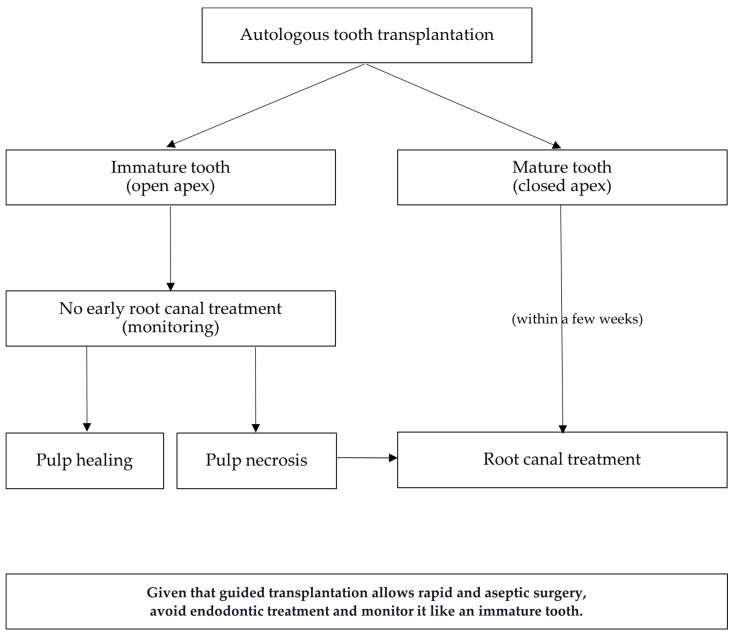
Decisional algorithm for the endodontic management of transplanted teeth.

After two weeks, the sutures and the splint were removed according to International Association for Dental Traumatology (IADT) treatment guidelines for avulsed permanent teeth replanted immediately [10]. The tooth had mobility but presented a physiological probing. In mature transplanted teeth, root canal treatment is performed 2 to 12 weeks after transplantation; on the contrary, immature transplanted teeth are monitored following the decisional algorithm shown in Figure 3. 

In this case, given the speed with which the transplantation was performed (approximately 1–2 s of extra-alveolar time), it was decided not to perform the endodontic treatment and to monitor the healing process in order to detect and manage any potential complications like infection, resorption, or ankylosis: clinical and radiograph controls at 2 weeks and 1, 3, 6, 12 (Figure 1C), 24, and 36 months (Figure 1D) showed a perfectly healed tooth.

**Figure 4:**
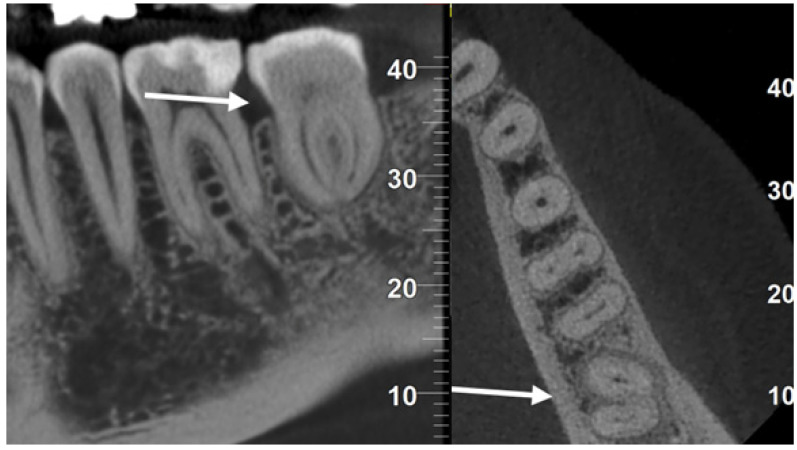
CBCT images at five years showed complete healing even though the transplanted donor tooth (arrows) was not endodontically treated.

After five years, the patient underwent a CBCT for other reasons. It was possible to exclude the presence of peri-radicular lesions or resorptions, confirming complete healing without complications, even without root canal treatment in a mature tooth (Figure 4).

In addition, the orthodontic forces applied pre-operatively triggered an inflammatory/repair activity that allowed the trophism of the PDL of the donor tooth to increase. Dental transplantation’s success derives from the PDL cells’ reparative capacity, so an already responsive periodontal ligament should improve its healing capacity.

Another significant result was patient satisfaction with avoiding implant rehabilitation.

## 4. Discussion

The most important considerations for successful tooth transplantation are preserving healthy PDL cells and good tissue adaption. These factors are related to surgical aspects, including the number of “fitting attempts” of the donor tooth, the distance between the recipient alveolus and the root of the donor tooth, the extra-alveolar time, the skill of the surgeon, and the atraumatic extraction of the donor tooth [6,11,12]. Different methods, including 3D radiographic imaging and 3D-printing technologies, have been proposed to update and facilitate the surgical technique to achieve greater success rates after the intervention [13]. Teeth replica models fabricated pre-operatively should be used as a fitting tester during recipient site preparation. Using these models enables the preparation of the neo-alveolus before the explantation of the donor tooth. Moreover, this may reduce the extra-alveolar time of the grafts and minimize the number of fitting attempts, thus preventing injury of PDL cells or pulp [11,14]. However, these methods still require free-hand preparation of the recipient site. 

Different protocols have been described for receiving socket remodeling. Anssari Moin et al. proposed a method without free-hand preparation, using a custom-printed osteotome as an alternative to implant drills, which reduced the number of CARP model fitting attempts. These authors reported results similar to those achieved by implant-guided surgery when comparing superimposed images of the pre-operative planning and the final donor tooth position [13]. Nevertheless, given that osteotomes have been associated with impaired bone healing when used in mandibular bone, this approach can only have a relevant role in cases of low bone density, as in the maxillary arch, and in the mandible, implant-like bur preparation of the recipient alveolus is preferable [7,8,15]. 

Thanks to the proximity of the recipient site and the preparation carried out, the extra-alveolar time was practically nil. For this reason, it is possible to consider that no contamination of the donor tooth and therefore no septic necrosis of the pulp occurred, allowing endodontic treatment to be omitted. According to Dioguardi et al., endodontic treatment of transplanted teeth with complete root formation must be carried out within 1–2 weeks from the surgery in order to prevent infection of the pulp from spreading from the periapical area and consequent inflammatory root resorption. In fact, only 15% of teeth with a closed apex are revitalized following the autotransplantation procedure, in contrast to 96% of teeth with an open apex [16]. On the other hand, Yu et al. reported an age-based treatment protocol: for patients over the age of 20, elective root canal treatment was provided; for younger patients, the vitality was monitored, and the treatment was performed if necrosis occurred [17]. Another study on completely or near-completely formed teeth showed that 29 out of 41 molars maintained vitality over the entire follow-up period (an average of eight years) [18]. Boschini et al. proposed an innovative approach with intra-operative apicoectomy without subsequent orthograde endodontic treatment as an alternative to early root canal treatment in mature teeth [19].

In the present case, at the five-year follow-up visit, the transplanted tooth did not show any sign of periapical reaction or resorption, demonstrating the reliability of the atraumatic extraction and preparation of the recipient socket. Moreover, no root canal treatment was necessary, probably due to the maintenance of the asepsis of the endodontic spaces during the transplantation. We do not know if pulp tissue is still vital or if it underwent aseptic necrosis and has been replaced by PDL-like tissue, as occurs in pulp revitalization [20]. Still, the goal, in this case, was to obtain healing in a mature donor tooth without endodontic treatment. 

## 5. Limitations

Autologous tooth transplantation can offer several advantages but there are also limitations and challenges associated with this procedure. Not all teeth are suitable for transplantation. The donor tooth must be healthy and easy to extract (no complex roots), have a similar root shape and size to the recipient site, and be extracted carefully to preserve its viability. The recipient site must have adequate bone support and healthy surrounding tissues for successful integration of the transplanted tooth. Patients must be informed about risk of root resorption, risk of infection at both the donor and recipient sites (which can compromise the success of the transplantation), the limited success rate compared to alternative treatments (dental implants or other prosthetic solutions), the need for orthodontic or restorative treatment, and the need for long-term monitoring to assess the health and stability of the transplanted tooth [21,22,23,24,25,26,27,28,29,30].

While computer-guided surgery could reduce the complication rates of autologous tooth transplantation and avoid endodontic treatment, it should be proposed in selected cases and it is essential to discuss with the patient the potential benefits and limitations.

## 6. Conclusions

Guided surgery appears to be a reliable option in tooth autotransplantation, helping to minimize the extra-alveolar time of donor teeth and recipient socket trauma, particularly in dense mandibular bone. Further studies are necessary to validate the technique proposed in this case report.

## Figures and Tables

**Table 1:** PRICE 2020 flowchart.

42-year-old woman
Slight pain at the second left lower molar (3.7)
The patient signed informed, valid consent for radiographic investigation
Medical history: no significant diseases declared by the patient
Previous dental history: the patient had conservative restorations and root canal therapy on an upper premolar. The sore tooth had already been restored
Clinical findings: teeth in good health except for the sore tooth where a subgingival cavity could be probed
Vitality test (thermal) positive, percussion test positive, intraoral X-ray, CBCT
Differential diagnosis: cervical invasive root resorption or penetrating cervical carious lesion
Definitive diagnosis: cervical invasive root resorption
Treatment options: (1) extraction and implant rehabilitation; (2) autotransplantation of 3.8 in 3.7
The patient signed informed, valid consent for autotransplantation
Treatment performed: guided tooth autotransplantation
5-year follow-up
Follow-up assessment methods: intra-oral X-rays and CBCT
Treatment outcome: complete healing without root canal treatment, even if the donor tooth was mature
Patient perspective: satisfaction with having a natural tooth instead of an implant
Conclusion: guided tooth autotransplantation was successful in avoiding root canal treatment
Funding details: none
Conflict of interest: none

## Data Availability

The data presented in this study are available on request from the corresponding author.

## Abbreviations

3D	three-dimensional
bds	two times a day (bis die sumendum)
CARP	computer-aided rapid prototyping
CBCT	Cone Beam Computerized Tomography
IADT	International Association for Dental Traumatology
*ii*	two tablets (duos doses)
PDL	periodontal ligament
STL	STereo Lithography interface format
tid	three times a day (ter in die)
